# Two-stage oxygen delivery for enhanced radiotherapy by perfluorocarbon nanoparticles: Erratum

**DOI:** 10.7150/thno.42420

**Published:** 2020-02-04

**Authors:** Zaigang Zhou, Baoli Zhang, Haoran Wang, Ahu Yuan, Yiqiao Hu, Jinhui Wu

**Affiliations:** 1 State Key Laboratory of Pharmaceutical Biotechnology, Medical School of Nanjing University & School of Life Sciences, Nanjing University, Nanjing 210093, China; 2 Institute of Drug R&D, Nanjing University, Nanjing 210093, China; 3 Jiangsu Provincial Key Laboratory for Nano Technology, Nanjing University, Nanjing 210093, China

In our paper^1^ and the supplementary materials, Figure 4F and Supplementary Figure 3A should be corrected as the following Figure A1 and Figure A2.

## Figures and Tables

**Figure A1:**
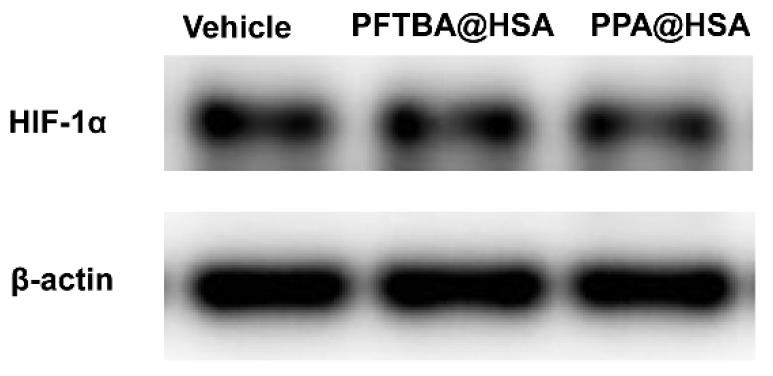
(Figure 4F). Representative western blot image to analyze HIF-1α expression in tumors from Vehicle and PFC@HSAs treated mice.

**Figure A2:**
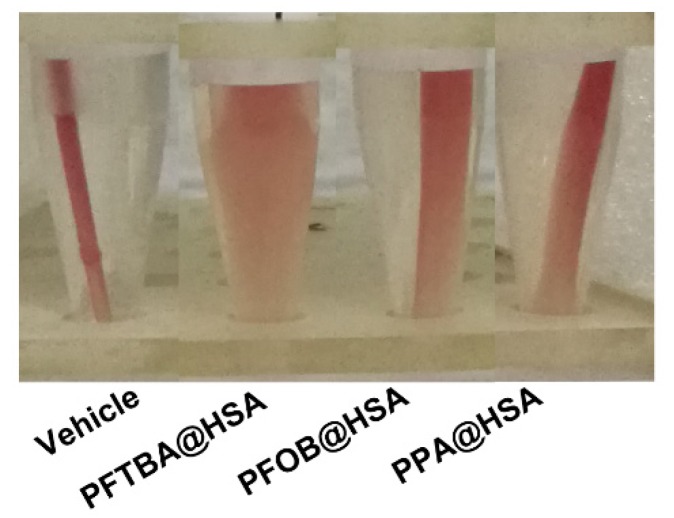
(Supplementary Figure 3A). Representative image of blood retraction after different PFC@HSAs treatments.

## References

[1] Zhou Z, Zhang B, Wang H, Yuan A, Hu Y, Wu J (2018). Two-stage oxygen delivery for enhanced radiotherapy by perfluorocarbon nanoparticles. Theranostics.

